# Effects of shallot (*Allium ascalonicum*) powder supplementation on growth, intestinal morphology, immune stimulation, and intestinal bacteria in broiler chickens

**DOI:** 10.14202/vetworld.2024.2338-2346

**Published:** 2024-10-17

**Authors:** Benyapha Surasorn, Peerapol Sukon, Pairat Sornplang

**Affiliations:** 1 Division of Animal Science, Faculty of Agriculture and Agricultural Industry, Surindra Rajabhat, University, Surin, 32000, Thailand; 2 Division of Veterinary Public Health, Faculty of Veterinary Medicine, Khon Kaen University, Khon Kaen, 40002, Thailand; 3 Faculty of Veterinary Medicine; Research Program on Toxic Substances, Microorganisms and Feed Additives in Livestock and Aquatic Animals for Food Safety, Khon Kaen University, Khon Kaen, 40002, Thailand

**Keywords:** broiler chickens, food security, immune stimulation, intestinal bacteria, intestinal morphology, shallot

## Abstract

**Background and Aim:**

Antimicrobial resistance in poultry farms is a significant global public health concern that has led farmers to explore alternative antibiotics, such as prebiotics in poultry production. This study aimed to examine the antimicrobial activity of ethanolic shallot extract (ESE) and the effects of adding shallot powder (SP) to broiler feed on broiler growth, immune response to Newcastle disease virus (NDV) vaccination, and gastrointestinal tract bacteria.

**Materials and Methods:**

We determined the antimicrobial effects of ESE against *Escherichia coli* O157:H7 (EOH) and *Lactobacillus acidophilus* TISTR 2365 (L2365) using the agar well diffusion method. We used a complete randomized design to assign 120 1-day-old Arbor Acre chicks to six groups with four replicates of five broiler chickens over 42 days. The treatment groups were as follows: T1-basal diet (B) + NDV vaccination (positive control), T2-B (negative control), T3-B + 2 g SP per kg of feed, T4-B + 2 g SP per kg of feed + NDV, T5-B + 4 g SP per kg of feed, and T6-B + 4 g SP per kg of feed + NDV.

**Results:**

The minimum inhibitory concentrations of ESE on EOH and L2365 were 62.50 and 125.00 mg/mL, respectively. The body weight gain, average daily gain, and feed conversion ratio in the 4 g SP of T5 and T6 groups were significantly improved compared with the other groups (p < 0.05). The immune organ (IO) and thymus gland weights in the T4 group were significantly greater than those observed in the positive and negative control groups (p < 0.05). The IO weights of the bursae of Fabricius and spleen tended to be greater in the T4 group than in the other groups. T5 group broilers had the highest ratio of villus height to crypt depth. The humoral immunity titers against NDV vaccination were improved in the SP-supplemented groups compared with the non-supplemented groups (p > 0.05). SP supplementation reduced the levels of coliform (p < 0.05) and E. coli in the broiler intestine by adding 4 *g* of SP per kg of feed. However, L2365 was more tolerant to ESE *in vitro* and tended to increase in line with increased SP levels.

**Conclusion:**

ESE showed strong antimicrobial activity to reduce harmful bacteria, and SP supplementation may exhibit prebiotic effects to increase broiler chicken growth, immunity, and microbial balance.

## Introduction

Broiler chicken rearing is widely practiced in Thailand and involves small- and large-scale agricultural operations. Consequently, chicken meat is prevalent and readily available in the Thai market; however, broiler chickens can potentially harbor pathogenic bacteria, such as *Salmonella* [1] and *Enterococcus* [2], which can be transferred to humans who consume contaminated chicken meat.

Farmers routinely use antibiotics to combat these pathogens; however, this practice raises concerns about developing antibiotic-resistant bacteria. One potential strategy to address these concerns involves using organic substances – including probiotics, prebiotics, symbiotics, and medicinal plants – to manage infectious diseases in broiler chickens rather than relying solely on antibiotics [3].

Thailand cultivates various tuber plants known for their prebiotic properties, such as onion, shallot, and garlic. These plants also contain short-chain sugars such as fructo-oligosaccharide (FOS) and inulin [4], which nourish beneficial microorganisms in an animal’s gastrointestinal tract (GIT). Prebiotics can alter GIT histomorphology or mucosa and improve its absorption ability in poultry [3]. Specifically, shallots (*Allium ascalonicum*) are widely consumed in Thailand. The Thai province of Sisaket is renowned as a significant cultivation region for shallots due to its dark red outer layer, purple inner layer, and pungent aroma [5].

Thai shallot is also frequently exported to Malaysia, Vietnam, and Singapore [6]. Many studies have reported that shallots contain phenolic compounds and flavonoids, highlighting their antioxidant and antimicrobial properties [7, 8]. An *et al*. [9] reported that incorporating onion (*Allium cepa*) extract into chicken diets improved growth performance and meat quality.

Despite these preliminary findings, the effects of shallot powder (SP) supplementation on broiler chickens’ immune systems, GIT morphology, and bacterial populations are limited. This study analyzed the influence of SP supplementation on broiler chicken growth, GIT morphology, immune response, and GIT microbiota composition.

## Materials and Methods

### Ethical approval

The use of birds in this study was approved under the permissions and guidelines of the Institutional Animal Care and Use Committee of Khon Kaen University, Thailand (Permission record no. IACUC-KKU-1/65).

### Study period and location

The study was conducted during June 2023 and July 2023 in the Division of Animal Science, Faculty of Agriculture and Agricultural Industry, Surindra Rajabhat University, Surin, Thailand.

### Preparation of SP and ethanolic shallot extract (ESE)

This study used dry bulbs of shallots cultivated in Sisaket Province, Thailand. The Thai Ministry of Commerce’s Department of Intellectual Property has officially registered it as a geographical indication (GI 63100142). First, each shallot’s outer skin was removed. The shallots were rinsed with tap water 2–3 times and then air-dried. After preparation, the shallots were minced into small pieces and desiccated in a hot-air oven at 60°C. Afterward, the desiccated samples were finely ground and divided into two portions. One portion was sealed in a vacuum-sealed bag and stored at 4°C until use as a chicken feed mixed powder; the other portion was used to produce ESE.

The method we employed for ESE preparation consisted of two primary phases: maceration and extraction. The finely ground SP was dissolved in ethanol while maintaining a 1:2 ratio of shallots to 95% ethanol [10]. The mixture was steeped for 7 days with daily agitation and filtered through Whatman No. 1 filter paper. The next step involved removing the ethanol using a vacuum evaporator until the crude ESE increased in density; the ESE was then stored at −20°C before use.

### Percentage yield of the ESE

The percentage yield of the ESE was evaluated by comparing the average ESE quantity obtained with the initial substance volume using the methodology described by Ruksiriwanich *et al*. [11]. This comparison is achieved using the following formula:

Percentage yield = (ESE weight/Initial weight of powdered shallot) × 100

### Antimicrobial activity of ESE

The efficacy of ESE in suppressing indicator microorganisms was assessed using the agar well diffusion technique. To conduct the study, the indicator bacterium *E. coli* O157:H7 (EOH), which was obtained from the GITs of broiler chickens and preserved at the Faculty of Veterinary Medicine, Khon Kaen University, was cultured in nutrient broth according to method described by Campana *et al*. [12]. The density of the bacterial culture was standardized to 0.5 McFarland using a 0.85% saline solution, as previously described by Ammor *et al*. [13]. Afterward, 0.1 mL of the bacterial suspension was evenly distributed on nutrient agar using a sterilized cotton swab and dried by exposure to air, and a well with a diameter of 6 mm was obtained. Subsequently, 40 mL of the ESE was added to each well of the culture medium. The tested bacteria were then inoculated onto the media surface. Sterile distilled water and 30 μg of tetracycline paper disk (Thermo Scientific, USA) were placed in the central wells of a Petri dish, serving as the negative and positive controls, respectively. The Petri dishes were air-dried and incubated at 37°C for 24 h. The antibacterial efficacy of ESE was determined by measuring the inhibition zone, and the process was repeated three times to ensure consistent elimination of bacteria, as recommended by Halder *et al*. [14].

### Determination of the minimum inhibitory concentration (MIC) of ESE against indicator bacteria

Determining the MIC of ESE against indicator bacteria requires applying the broth dilution method in nutrient broth. The procedure involved the preparation of the ESE at a concentration of 500 mg/mL for experimental evaluation. The ESE was diluted using a two-fold technique at concentrations of 500, 250, 125, 62.5, 31.25, 15.62, 7.81, 3.90, and 1.95 mg/mL distributed across a 96-well plate. A bacterial suspension was then generated by adjusting the turbidity to a standard of 0.5 McFarland. The cohouse bacterial suspension was dispensed 1 mL into each ESE-containing well on the plate. The prepared wells were then incubated at 37°C for 24 h. The MIC was determined by identifying the wells where no bacterial proliferation was detected or where the culture medium in the well-remained unclouded. The control groups consisted of a positive nutrient-control broth incorporating bacteria and a standard antibiotic solution and a negative control without test bacteria. The resulting bacterial growth was meticulously observed, and the MIC was reported as mg/mL units.

### Diets and experimental animals

Arbor Acres broiler chickens (n = 120), mixed-sex and aged 1 day were randomly assigned to one of six experimental (Treatment, T) groups (each group replicated 4 times with five chickens) using a completely randomized design. All groups were fed a standard diet featuring a standard nutrient composition (Table-1), as recommended by the National Research Council [15]. Water was freely available *ad libitum* to all chicken groups. The specific feed requirements of each experimental group were as follows:

**Table-1:** Nutrient composition of basal diets.

Item	Starter	Grower
	
	1–21 days	22–42 days
Ingredient (kg)		
Corn	47.80	60.25
Soybean meal (44% crude protein)	28.55	18.00
Full-fat soybean	17.05	15.00
Rice bran oil	2.00	2.10
Choline chloride	0.10	0.10
Dicalcium phosphate (21% phosphorus)	1.80	1.80
Limestone	1.60	1.60
DL-methionine	0.30	0.30
L-lysine	0.15	0.15
Vitamin-mineral premix^1^	0.25	0.30
NaCl	0.40	0.40
Total	100.00	100.00
Nutrient composition		
Calculated^2^		
Metabolizable energy (kcal/kg)	3001.00	3100.00
Crude protein (%)	22.51	18.01
Calcium (%)	1.24	1.20
Available phosphorus (%)	0.74	0.70

1 supplied per kg of diet: Vitamin A, 1,500 IU; cholecalciferol, 200 IU; Vitamin E, 10 IU; riboflavin, 3.5 mg; pantothenic acid, 10 mg; niacin, 30 mg; cobalamin, 10 µg; biotin, 0.15 mg; folic acid, 0.5 mg; thiamine, 1.5 mg; pyridoxine, 3.0 mg; iron, 80 mg; zinc, 40 mg; manganese, 60 mg; iodine, 0.18 mg; copper, 8 mg; selenium, 0.15 mg.

2 Based on NRC recommendations [15]

- T1 cells received basal diet (B) plus Newcastle disease virus (NDV) vaccination (positive control)
- T2 received B (negative control)
- T3 received B plus SP supplemented with 2 g/kg feed
- T4 received B plus SP supplemented with 2 g/kg feed and NDV
- T5 received B plus SP (4 g/kg feed) and
- T6 received B plus SP at a feed ratio of 4 g/kg and NDV

We determined the initial weight of each chick and then sorted and randomly selected chicks with similar weights for distribution to each group. On 14 days of age, all experimental chicken groups were vaccinated. NDV was administered on 7 and 14 days based on the predetermined allocation within the experimental groups, which were not administered the vaccine on day 14 in the non-NDV groups, as previously mentioned regarding the T2, T3, and T5 groups. The chicks were maintained under suitable warmth provided by a light bulb ranging from 25 to 40 W (adjusted according to ambient temperature), fed twice daily in the morning and evening (at 07.00 AM and 4.30 PM) and exposed to light following the natural light cycle as recommended by the Ethics Committee.

### Growth

Over 6 weeks, all 120 chicks (20 chicks per group) were monitored. The following parameters were recorded: Body weight gain (BWG), feed intake (FI), and feed conversion ratio (FCR) every 3 weeks. The BWG was calculated by subtracting the initial body weight (g) from the final body weight (g). FI is measured in grams of the total chicken feed consumed over time. The FCR was measured in grams and was calculated as the final BWG divided by the total FI.

### GIT morphology

Eight 42-day-old chickens from each experimental group were euthanized through cervical dislocation. After this process, a 2-cm portion of the jejunum was investigated for the potential completed villi by excising the intestine at the halfway (location of Meckel’s diverticulum). The subsequent stage involves the longitudinal opening and placement of the intestine, with the villi positioned upward on a foam support. The specimen should be submerged in a 10% buffered neutral formalin solution in a plastic receptacle, as recommended by Awad *et al*. [16], to maintain cellular integrity before histological assessment. An Olympus BX50 microscope (Japan) with 20× optical magnification is used to capture images for histological examination of the small intestine, followed by image analysis software (ToupView version 3.7, ToupTek Photonics, China) [17] for additional examination. The villus height (VH) and crypt depth (CD) were evaluated, with 10 villi selected randomly per slide. Subsequently, the VH-to-CD ratio (VH: CD) was computed using the methodologies established by Gava *et al*. [18].

### Immune organ (IO) indices

The IO indices were calculated by weighing the thymus, spleen, and bursa as the organ weight (g)/body weight (kg) [19].

### Immune response to vaccination

After NVD vaccination, blood samples were collected from eight broiler chickens randomly selected from each group on days 21 and 35. The samples were obtained by puncturing the wing vein, and 2 mL of each chicken was taken and centrifuged at 3000× *g* for 10 min to facilitate serum separation. The collected serum samples were sent to an animal hospital, Faculty of Veterinary Medicine, Khon Kaen University, Thailand, to determine antibody titers to NDV using the hemagglutination inhibition (HI) test [20].

### The GIT bacterial population

Eight 42-day-old chickens from each group were randomly selected and sacrificed by cervical dislocation. GIT (ileum part) samples were collected to determine *Escherichia coli* and *Lactobacillus* spp. Counts using the spread and pore plate methods, respectively. GIT content (5 g) samples were mixed in 45 mL of buffered peptone water (BPW, Oxoid Ltd., Basingstoke, U.K.) and diluted tenfold. BPW at 10^3^–10^7^ m was poured with De Man–Rogosa–Sharpe (MRS) agar and incubated in 5% CO_2_ at 30°C for 48 h for *Lactobacillus* detection. The dilution was spread onto MacConkey (Oxoid Ltd., Basingstoke, U.K.) and Eosin Methylene Blue (EMB, Oxoid Ltd., Basingstoke, U.K.) agar plates and incubated at 37°C for 24 h. Pink and green-metallic sheen colonies from MacConkey agar and EMB agar, respectively, were selected and confirmed through biochemical tests of indole, methyl red, Voges-Proskauer, and citrate utilization tests for *E. coli* detection, according to Anyanwu *et al*. [21].

## Results

### Percentage yield of the ESE

**Figure-1:**
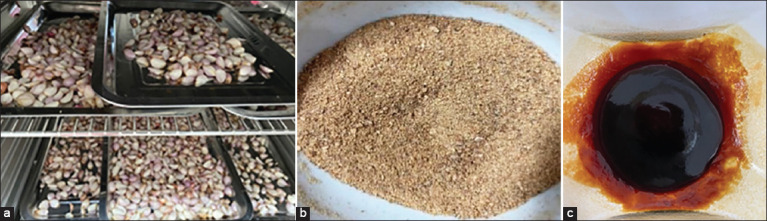
Shallot extraction steps: (a) Fresh sample for drying, (b) total powder, and (c) total crude extract.

After the conversion of fresh SP into ESE at a rate of 100 g of fresh shallots yielding 16 g of ESE, a viscous dark brown liquid was derived from this process (Figure-1), yielding 87.50%.

### Antimicrobial activity of ESE

We found that it could hinder *E. coli* O157:H7 infection, resulting in an average inhibition zone diameter of 10.11 mm, which was smaller than that resulting from exposure to tetracycline. Moreover, ESE tended to inhibit EOH to a greater extent than L2365 (Table-2).

**Table-2:** Inhibition zone of the antimicrobial activities of the shallot crude extract.

Item	Inhibition zone (mm)^1^	

	*Lactobacillus*	*E. coli* O157:H7
Shallot	2.08 ± 0.13	10.11 ± 0.38
Distilled water	0.00 ± 0.00	0.00 ± 0.00
Tetracycline	15.56 ± 0.23	12.94 ± 0.25

Concentration of the shallot crude extract=500 mg/mL.

1 Inhibition zone is expressed as mean ± SD in triplicate. *E. coli*=*Escherichia coli*

### MIC of ESE against indicator bacteria

The MICs of ESE against EOH and L2365 are listed in Table-3.

**Table-3:** Determination of the MIC of the shallot extract against indicator bacteria.

Bacteria	MIC (mg/mL)
*E. coli* O157:H7	62.50
*Lactobacillus acidophilus* TISTR 2365	125.00

MIC=Minimum inhibitory concentration,*E. coli*=*Escherichia coli*

### Growth

Adding SP to the diet of broilers in this study affected their growth performance. No statistically significant differences (p > 0.05) in the average daily gain (ADG) were noted among the experimental groups during the initial 0–3 weeks. Nevertheless, a significant difference (p < 0.05) in ADG was detected between the experimental groups at 3–6 weeks of age. Specifically, the T5 and T6 groups exhibited the highest ADGs at 86.75 and 77.51 g, respectively. Throughout the entire 0–6-week period, the ADG significantly differed (p < 0.05), with the T5 and T6 groups exhibiting the highest values of 65.75 and 59.11 g, respectively, compared with the other groups. There were no significant differences (p > 0.05) in the average FI among the experimental groups at 0–3 weeks and 3–6 weeks of age.

However, during the 3–6-week period, the FI showed notable differences between the T1, T2, T4, and T5 groups and the T3 group (p < 0.05). Over the entire 0–6 weeks of rearing phase, the T1, T4, and T5 groups displayed notably greater FI than the T3 group (p < 0.05). The FCR did not differ significantly between weeks 0 and 3 weeks (p > 0.05) among the experimental groups, which varied from 1.51 to 1.64. Conversely, at 3–6 weeks, the T2, T3, T4, T5, and T6 groups exhibited substantially lower FCRs than the T1 group (p < 0.05). Moreover, at 0–6 weeks of age, the T2, T5, and T6 groups presented significantly lower FCRs than the other groups (p < 0.05), with values of 1.86, 1.71, and 1.81, respectively, as shown in Table-4.

**Table-4:** Effects of shallot powder supplementation on broiler growth performance.

Parameters	Treatment						p-value	SEM^1^

	T1	T2	T3	T4	T5	T6		
Initial weight (g/bird)								
	0.040	0.041	0.041	0.040	0.041	0.041	0.54	0.120
BWG (g/bird)								
0–3 weeks	920.06	930.50	879.25	879.25	906.79	864.44	0.65	12.63
3–6 weeks	1298.19^c^	1517.50^bc^	1468.00^b^	1526.75^bc^	1818.52^a^	1631.06^ab^	0.01	45.03
0–6 weeks	2218.25^c^	2448.00^bc^	2347.25^bc^	2406.00^bc^	2725.31^a^	2495.50^ab^	0.01	43.84
ADG (g/bird/d)								
0–3 weeks	43.81	44.31	41.87	41.43	43.18	41.16	0.653	0.60
3–6 weeks	61.82^c^	72.26^bc^	69.90^bc^	72.70^bc^	86.60^a^	77.67^ab^	0.01	2.14
0–6 weeks	52.82^c^	58.29^bc^	55.89^bc^	57.29^bc^	64.89^a^	59.42^ab^	0.01	1.04
Feed intake (g/bird/d)								
0–3 weeks	1453	1436	1412	1501	1459	1457	0.09	0.008
3–6 weeks	3154^a^	3124^a^	2984^b^	3169^a^	3163^a^	3073^ab^	0.05	0.02
0–6 weeks	4607^a^	4560^ab^	4396^b^	4670^a^	4623^a^	4531^ab^	0.03	0.02
Feed conversion ratio								
0–3 weeks	1.51	1.47	1.53	1.64	1.53	1.62	0.35	0.02
3–6 weeks	2.50^a^	2.06^a^	2.03^a^	2.08^a^	1.77^b^	1.88^ab^	0.01	0.06
0–6 weeks	2.09^a^	1.86^b^	1.87^ab^	1.94^ab^	1.71^b^	1.81^b^	0.03	0.03

^a,b^Treatment means with different superscripts within the same row are significantly different at p *<* 0.05.

1 Standard error of the mean (n = 20, from 4 replicate cages of 5 birds each). Treatments: T1=Basal diet (B) + Newcastle disease virus (NDV) vaccination, T2=B, T3=B + 2 g of shallot powder/kg of feed, T4=B + 2 g of shallot powder/kg of feed + NDV, T5=B + 4 g of shallot powder/kg of feed, and T6=B + 4 g of shallot powder/kg of feed+NDV, SEM=Scanning electron microscope, BWG: Body weight gain, ADG: Average daily gain

### GIT morphology

**Figure-2:**
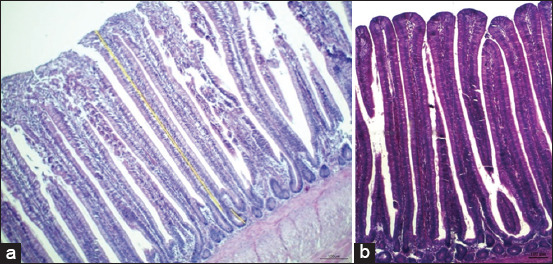
A representative photomicrograph of 40× magnification hematoxylin and eosin-stained jejunal segments of broiler chickens. The villi height of 42-day-old broiler chickens in the (a) non-supplemented shallot powder (SP) treatment group was lower than that in (b) the supplemented SP group.

We also examined the impact of incorporating SP into the diet of broiler chickens on the ratio of VH to CD in the jejunum on 42 days of age. A statistically significant between-group difference (p < 0.05) was detected between the supplemented and control groups. Among the supplemented groups, T5 exhibited the highest VH at 798 μm, followed by T4 and T6 at 771 μm and 752 μm, respectively. Conversely, Group T2 exhibited the lowest value at 527 μm. The CD of the intestines had lengths in the order of 52 μm, 62 μm, 65 μm, 70 μm, and 72 μm for the T2, T3, T6, and T4 groups, with T4 being equal to the values for the T1 and T5 groups, respectively (Table-5 and Figure-2).

**Table-5:** Effects of shallot powder supplementation on villus height and jejunal crypt depth in 42-day-old broilers.

Treatment	Villus height (VH) (µm)	Crypt depth (CD) (µm)	VH: CD ratio
T1	664^b^	70^ab^	9.49
T2	527^c^	52c	10.13
T3	661^b^	62^bc^	10.66
T4	771^ab^	70^ab^	11.01
T5	798^a^	72^a^	11.08
T6	752^ab^	65^ab^	11.57
p-value	0.0001	0.002	0.24
SEM1	0.018	0.002	0.32

^a,b^Treatment means with different superscripts within the same column are significantly different at p *<* 0.05. ^1^Standard error of means (n = 8, each replicate consisted of two birds). Treatments: T1=Basal diet (B) + Newcastle disease virus (NDV) vaccination, T2=B, T3=B + 2 g of shallot powder/kg of feed, T4=B + 2 g of shallot powder/kg of feed+NDV, T5=B + 4 g of shallot powder/kg of feed, and T6=B + 4 g of shallot powder/kg of feed + NDV. VH: CD: Villus height-to-crypt depth ratio

### IO indices and immune response to vaccination

The effects of SP supplementation on broiler chicken organs were assessed on 42 days of age. The glands of the thymus ranged in size from 1.70 to 2.10. The supplemented groups exhibited greater values than the non-supplemented groups (p < 0.05). The bursa gland value ranged from 1.46 to 2.01. The T4 group had the highest bursa gland weight, 2.01 g (p < 0.05), as shown in Table-6. We evaluated the immune response to NDV in broiler chicken diets supplemented with SP on 21 and 35 days of age. Blood was drawn from randomly selected chickens after 7 and 21 days of vaccination; however, the results indicated no significant effect of SP on NDV (Table-7).

**Table-6:** Effect of shallot powder supplementation on the relative immune organs of broilers.

Treatment	Immune organ indexes^1^		

	Thymus	Bursa of Fabricius	Spleen
T1	1.70	1.46^b^	1.43^ab^
T2	1.70	1.50^b^	1.40^ab^
T3	1.76	1.70^ab^	1.45^ab^
T4	2.10	2.01^a^	1.76^a^
T5	1.91	1.73^ab^	1.33^b^
T6	2.03	2.00^a^	1.40^ab^
p-value	0.53	0.04*	0.05
SEM2	0.06	0.08	0.05

^a,b^Treatment means with different superscripts within the same column are significantly different at p *<* 0.05.

* The immune organ weights among the treatment groups were significantly different (p < 0.05).

1 The immune organ index is calculated by weighing the organ weight (g)/body weight (kg). ^2^Standard error of means (n = 8, each replicate consisted of two birds). Treatments: T1=Basal diet (B) + Newcastle disease virus (NDV) vaccination; T2=B, T3=B + 2 g of shallot powder/kg of feed, T4=B + 2 g of shallot powder/kg of feed+NDV, T5=B + 4 g of shallot powder/kg of feed, and T6=B + 4 g of shallot powder/kg of feed + NDV

**Table-7:** Effect of shallot powder supplementation on HI titers of Newcastle disease virus in 21- and 35-day-old broiler chickens.

Treatment	HI titer	

	21 day	35 day
T1	3.00	3.67
T2	2.33	3.33
T3	2.33	3.00
T4	3.00	3.33
T5	3.00	4.00
T6	3.00	3.67
p-value	0.45	0.57
SEM^1^	0.30	0.22

1 Standard error of the mean (n = 8, each replicate consisted of two birds). Treatments: T1=Basal diet (B) + Newcastle disease virus (NDV) vaccination, T2=B, T3=B + 2 g of shallot powder/kg of feed; T4=B + 2 g of shallot powder/kg of feed + NDV; T5=B + 4 g of shallot powder/kg of feed; and T6=B + 4 g of shallot powder/kg of feed + NDV. HI: Hemagglutination inhibition

### The GIT bacterial population

SP supplementation altered the levels of *E. coli* and *Lactobacillus* spp. measured in feces samples obtained after 42 days of age across all experimental groups. The bacterial levels ranged from 2.26 to 3.30 log CFU/g for *E. coli* and 7.96 to 10.51 log CFU/g for *Lactobacillus* spp. Although reduced *E. coli* levels were observed in Groups T3, T4, T5, and T6, the change was not significant (p > 0.05).

Conversely, *Lactobacillus* spp. levels tended to increase in Groups T5 and T6, with values of 9.88 and 10.51 log CFU/g, respectively; however, the difference was not statistically significant (p > 0.05; Table-8). The coliform counts varied from 3.40 to 7.40 log CFU/g, indicating a substantial decrease in coliform (p < 0.05) in the supplemented compared with the non-supplemented groups.

**Table-8:** Effect of shallot powder supplementation on the ileal bacterial population in 42-day-old broilers.

Treatment	Bacterial concentration (log cfu/g)		

	*E. coli*	Coliform	*Lactobacillus* spp.
T1	3.16	7.40^a^	8.82
T2	3.30	7.23^a^	7.96
T3	2.56	4.56^b^	9.47
T4	2.95	4.36^b^	9.82
T5	2.26	2.93^b^	9.88
T6	2.60	3.40^b^	10.51
p-value	0.09	0.02	0.55
SEM^1^	0.12	0.48	0.58

^a,b^Treatments means with different superscripts within the same column are significantly different at p < 0.05.

1 Standard error of the mean (n = 8, each replicate consisted of two birds). Treatments: T1=Basal diet (B) + Newcastle disease virus (NDV) vaccination, T2=B, T3=B + 2 g of shallot powder/kg of feed; T4=B + 2 g of shallot powder/kg of feed+NDV; T5=B + 4 g of shallot powder/kg of feed; and T6=B + 4 g of shallot powder/kg of feed + NDV. *E. coli*=*Escherichia coli*

## Discussion

The MICs of ESE against EOH and L2365 were 62.50 and 125.00 mg/mL, respectively. Our results illustrate the suppressive effects of unrefined ESE on *E. coli* proliferation compared with *Lactobacillus*. Consequently, the microbial community composition in the GIT can be modified, resulting in beneficial effects on poultry health.

This investigation revealed an increase in the FCR in the groups supplemented with shallot compared with the non-supplemented group (p < 0.05). Our results revealed increased ADG in the supplemented compared with the non-supplemented groups. However, the ADG observed in this study was inferior to that reported by Omar *et al*. [22], which could be attributed to variations in the bioactive composition of *A. ascalonicum* and *A. cepa*.

*A. cepa*, commonly called “onion,” is rich in bioactive quercetin, flavonols, sulfur compounds, and essential nutrients such as carbohydrates, proteins, and vitamins. Conversely, *A. ascalonicum*, known as “shallot,” harbors active constituents such as flavonoids, saponins, phenols, tannins, and alkaloids, which underpin its antibacterial properties [23]. Therefore, *A. cepa* supplementation is associated with increased nutrient intake in broilers and may increase ADG levels more than *A. ascalonicum* supplementation. However, we found that SP supplementation was more effective at inhibiting *E. coli* and increasing *Lactobacillus* spp. in the GIT of broiler chickens; growth was less affected. This result may be due to the stronger antimicrobial and prebiotic properties of *A. ascalonicum* [8] than those of *A. cepa*.

We used histomorphology to examine the effects of SP supplementation on the jejunum’s VH or CD in 42-day-old broilers. We noted distinctly elongated villi and increased CD in the jejunums of supplemented broilers compared with their non-supplemented counterparts (p < 0.01; Table-4 and Figure-2). This study revealed a positive correlation between increased VH and CD in the GIT and increased ADG. This correlation could be attributed to improved feed digestion and absorption by broilers. These findings agree with those of Omar *et al*. [22], who demonstrated that adding *A. cepa* at concentrations ranging from 1 g/kg to 3 g/kg increased VH and CD in the jejunum, with improved ADG in broilers.

Small intestinal epithelial cells are critical for nutrient absorption and digestion. These cells originate from the Lieberkühn crypt and migrate upward along the villi, shedding into the lumen of the small intestine on reaching the villus tip [24]. Adedokun and Olojede [25] suggested that GIT infection or inflammation can shorten and thicken villi, potentially reducing nutrient absorption. Our broiler supplementation demonstrated increases in VH, potentially due to various compounds in the shallots, including flavonoids and quercetin, which are known for their antioxidant and anti-inflammatory properties [26]. Abdulkareem *et al*. [27] found that adding garlic and onion (2.5%–5%) to broiler feed increased jejunal length, FI, and ADG.

Similarly, our study revealed positive effects on jejunal villus length, FI, and ADG although chicken feed was supplemented with only shallot. This result can be explained by garlic and onion (or shallot) having antioxidant and antimicrobial properties. However, garlic appears to have a more potent antimicrobial activity than onion due to its allicin content [28]. Onion species, such as *A. cepa* and *A. ascalonicum*, have stronger phenolic compounds and antioxidant properties than garlic [22, 23].

Regarding our study’s VH: CD ratio findings, no significant difference was detected between supplemented and non-supplemented broilers. This finding is in contrast with the results of Omar *et al*. [22], who suggested that including SP in a broiler diet decreased the VH: CD ratio compared with that in non-supplemented animals. These findings suggest that broilers who consumed a basal diet without SP supplementation had increased jejunal CD without a proportional increase in VH. This approach reduced the VH: CD ratio and nutrient absorption within the GIT.

Our results demonstrated an enhanced immune response in broiler supplementation, as evidenced by a significant increase in immune-related organ weights, such as the bursa of Fabricius. Compared with the positive and negative control groups, we also observed effects on antibody levels following NDV vaccination in 35-day-old broilers supplemented with SP. The bursa, which is central to the cellular immune system in birds, may be stimulated by the FOS component of SP supplementation in this study, similar to that reported by Song *et al*. [29]. Thus, SP supplementation at 4 g/kg feed led to higher average HI titers of NDV, regardless of vaccination status, than the other treatments. In addition, higher HI titers were more likely to be observed in 21-day-old broilers. No significant difference in average HI titers was noted between supplemented and non-supplemented broilers. However, those supplemented with 4 g/kg of SP had an HI titer for NDV of at least 3 log_2_, which is a sufficient concentration for immune stimulation and prevention of NDV infection [30]. The uncertain antibody response to NDV observed in this study may be attributed to interference between antibodies from the vaccine and those transmitted from hens to chicks.

SP supplementation increased the abundance of *Lactobacillus* spp. while concurrently decreasing detrimental microorganism levels such as *E. coli* and *Coliform* bacteria, in the ileum of broilers. The bioactive components of shallot exhibited anti-*E. coli* properties both *in vitro* and in broilers in this study. These findings agree with those of Moldovan *et al*. [8], who reported that the MICs of shallot against Gram-positive bacteria such as *Staphylococcus epidermidis* and *Bacillus subtilis* vary in the range of 25–50 mg/mL and against Gram-negative bacteria such as *Mycobacterium* spp. at 100 mg/mL. Our study revealed that the MIC of shallot against EOH (62.5 mg/mL) was lower than that reported by Igbokwe *et al*. [31], who reported an MIC of 100 mg/mL. The antimicrobial properties of *Allium* species can vary depending on the bioactive compounds, extraction processes, and microbial species used [8]. Tuber plants commonly found in Thailand, including onion, shallot, and garlic, contain varying concentrations of FOS and inulin [4]. Our results suggest that shallots contain FOS and inulin, which are prebiotics for beneficial microorganisms such as *Lactobacillus* spp.

## Conclusion

The supplementation of onion-related species such as shallot (*A. ascalonicum*) in broiler feed confers advantages to the health of broilers. SP supplementation resulted in strong antioxidant and antimicrobial activities, including improved jejunal VH, BWG, ADG, and FCR. We also noted the inhibition of pathogenic bacteria such as coliform and EOH, whereas beneficial microorganisms such as *Lactobacillus* spp. were promoted within GIT tissue samples obtained from broiler chickens. Moreover, 4 g/kg SP supplementation enhanced immune responsiveness, elevated HI titers in response to NDV vaccination on 35 days of age, and increased the relative weight of IOs like the bursa. These findings surpassed those observed in the control group. Importantly, we only tested two levels of SP supplementation, which could limit observable dose-response variations. Future research on animals could explore additional SP doses to optimize SP supplementation in broiler chicken diets.

## Authors’ Contributions

PS and BS: Conceptualized and designed the study. BS: Sample collection and microbiological culturing. PS and PSu: Statistical analyses. PS: Drafted the manuscript. PSu and PS: Revised the manuscript. All authors have read and approved the final manuscript.
